# 100 Years Ago

**Published:** 1983-07

**Authors:** 


### 100 Years Ago

### From the *Bristol Medico-Chirurgical Journal*, July 1883

**Volume 1, Number 1**
Page 1—*'The proofs of the existence of a Phthisical Contagion'* By R. Shingleton Smith
The author recalls that 'our late colleague, Dr. William Budd' startled the profession by announcing his belief that 'Phthisis would be found to be contagious' (Lancet 1867) and quotes his reasons. He refers to Koch's discovery (1882) of a peculiar micro-organism—delicate rod shaped bacilli with specific staining characteristics which he was able to cultivate artificially, inoculate into animals and produce tuberculous lesions. He summarises much other evidence and examines his own series of 77 cases in 49 of which he was able to demonstrate Koch's bacillus and he concludes 'If these observations be accurate there is only one conclusion, that these bacilli cause tuberculosis'.

Page 48—*'Clinical evidence against the contagiousness of Phthisis'* By E. Markham Skerritt
The author argues that even if the tubercle bacillus is associated with tuberculous lesions it may not be the cause—as smoke is not the cause of fire, and even if it be the cause it does not follow that the disease is contagious. He concludes that the contagiousness of Phthisis is as yet 'not proven'.

Page 71—*'The Cardiograph in Medicine'* by G. Munro Smith
The author describes an apparatus consisting of 2 drums with rubber diaphragms connected by a flexible tube. One is applied to the chest wall over the apex and the other has a lever attached to the diaphragm which records its movements on blackened paper.

Analysis of tracings showed characteristics for the various valvular lesions of the heart. He ends 'my only apology for bringing together and attempting to explain the above cardiograms is that so little has been done, especially in England, in what might become a fruitful field of clinical work'.

Page 136—The section *Medical Extracts* contains much of interest including a report, perhaps the first in the English Language of the *first cholecystectomy* in November 1882 by Langenbuch in Berlin. A T-shaped incision was made at the outer margin of the rectus muscle, the cystic duct was ligatured and the bladder cut away. The patient made a very rapid recovery, having got up in twelve days. At the end of four months he reported himself in perfect health.

From the *New York Medical Record* is quoted an account of a *Tape Worm Trap*—it was made of gold and had 'an elaborate interior economy', 'after being baited with a tempting morsel and attached to a string the instrument is swallowed, the unwary worm pushes in its head, is caught and dragged out by the string'. These two extracts only are quoted out of 11 others from the world literature of scarcely less interest.

